# Shape Matters: Comprehensive Analysis of Star-Shaped Lipid Nanoparticles

**DOI:** 10.3389/fphar.2020.00539

**Published:** 2020-04-30

**Authors:** Shuwen Cao, Xiaodi Liu, Xiuling Li, Chunhao Lin, Wenyue Zhang, Chee Hwee Tan, Shunung Liang, Baoming Luo, Xiaoding Xu, Phei Er Saw

**Affiliations:** ^1^Guangdong Provincial Key Laboratory of Malignant Tumor Epigenetics and Gene Regulation, Sun Yat-sen Memorial Hospital, Sun Yat-sen University, Guangzhou, China; ^2^Medical Research Center, Sun Yat-sen Memorial Hospital, Sun Yat-sen University, Guangzhou, China; ^3^The Ultrasound Department, Sun Yat-sen Memorial Hospital, Sun Yat-sen University, Guangzhou, China; ^4^The First Clinical Medical School of Guangzhou University of Chinese Medicine, Guangzhou University of Chinese Medicine, Guangzhou, China

**Keywords:** star, shape, nanocarrier, nanoparticle, lipid, uptake, permeability

## Abstract

The research of lipid nanoparticles (LNPs) has been ongoing for more than three decades, and more research are still being carried out today. Being the first Food and Drug Administration (FDA)-approved nanomedicine, LNPs not only provide various advantages, but also display some unique properties. The unique lipid bilayer structure of LNPs allows it to encapsulate both fat-soluble and water-soluble molecules, hence enabling a wide range of possibilities for the delivery of therapeutic agents with different physical and chemical properties. The ultra-small size of some LNPs confers them the ability to cross the blood brain barrier (BBB), thus obtaining superiority in the treatment of diseases of the central nervous system (CNS). The ability of tumor targeting is one of the basic requirements to be an excellent delivery system, where the LNPs have to reach the interior of the tumor. Factors that influence tumor extravasation and the permeability of LNPs are size, surface charge, lipid composition, and shape. The effect of size, surface charge, and lipid composition on the cellular uptake of LNPs is no longer recent news, while increasing numbers of researchers are interested in the effect of shape on the uptake of LNPs and its consequential effects. In our study, we prepared three lipid nanostars (LNSs) by mixing phosphatidylcholine (PC) with different backbone lengths (C14:C4 or C16:C6 or C18:C8) at a 3:1 ratio. Although several star-shaped nanocarriers have been reported, these are the first reported star-shaped LNPs. These LNSs were proven to be safe, similar in size with their spherical controls (~100 nm), and stable at 37°C. The release rate of these LNSs are inversely related to the length of the lipid backbone. Most importantly, these LNSs exhibited greatly enhanced cellular uptake and *in vivo* tumor extravasation compared with their spherical controls. Based on the different uptake and pharmacokinetic characteristics displayed by these LNSs, numerous route formulations could be taken into consideration, such as *via* injection or transdermal patch. Due to their excellent cellular uptake and *in vivo* tumor accumulation, these LNSs show exciting potential for application in cancer therapy.

## Introduction

Current strategies for cancer therapy include chemotherapy, surgery, angiogenesis, and monoclonal antibody therapy. However, tumors either quickly become resistant to these treatment modalities, or severe side effects occur, leading to discontinuity of the treatment or significant degradation to quality of life (Zugazagoitia et al., 2016). Along with the advent of nanoparticles, cancer diagnosis and therapy has emerged into a new era. The enhanced permeation and retention (EPR) effect was first reported by Maeda and Matsumura in 1986, stating that the blood vessels in tumor areas are formed fortuitously and thus inconsecutively due to the rapid growth of blood vessels stimulated by growth factors secreted by tumor cells (Matsumura and Maeda, 1986). The pores between endothelial cells allow nanoparticles to passively accumulate in tumors, which provides a new avenue for tumor targeting therapy. Over the past decades, various nanomedicine have been designed and approved to be reliable for cancer therapy, among them, lipid nanoparticles (LNPs) are the most time-honored nanomedicine and also the pioneer to be applied clinically (Shi et al., 2017). Liposomal doxorubicin modified by poly(ethylene glycol) (Doxil) was the first nanomedicine approved by the US Food and Drug Administration for cancer therapy. As the oldest and still widely researched nanomedicine, LNPs provide various advantages that other nanomedicines offer, such as specificity (Flayhan et al., 2018) and multi-functionality (Han et al., 2014; Ma et al., 2015). Meanwhile, LNPs possess some unique advantages compared with other nanomedicine. LNPs have an aqueous inner part, and a surrounding of one or more concentric lipid bilayers. This unique structure allows LNPs to encapsulate both fat-soluble and water-soluble molecules, hence providing a wide range of possibilities to deliver therapeutic reagents with different physical and chemical properties (Khan et al., 2008). Unlike polymeric nanoparticles, the diameter of LNPs range from 1 to 200 nm. Due to their sub-micron ultra-small size, LNPs are not only physically stable in structure, but are also able to pass through the blood-brain barrier (BBB) to enter the central nervous system (CNS), gaining widespread attention among various nanoparticles for the treatment of CNS diseases (Saw et al., 2017a; Mendes M et al., 2018). Other superiorities of LNPs include facilitated industrial production, suitable bioavailability (Din et al., 2015), biocompatibility (Eiras et al., 2017), improved drug absorption (Wang et al., 2017), and delayed dissolution (Xu et al., 2010).

To be an excellent delivery system, LNPs have to reach the core of tumors. Clearly, passively targeting the tumor by the EPR effect is insufficient, as the physical and chemical properties of the LNPs itself may affect its permeability. The extravasation ability of the nanocarrier is largely dependent on its size. The accumulation of nano-scale particles in the tumor tissue is not only due to the EPR effect, but also size of nanoparticles shows significant influence on the cell penetration. Although there is no conclusive ideal size for maximal cellular uptake of nanocarriers, research has reported that nanoparticles with certain sizes exhibit a better ability to be internalized, for example 50 nm Au nanoparticles show better permeability into HeLa cells compared to any other sizes (Chithrani et al., 2006). The surface charge of the nanocarrier accounts for another well-established factor in its ability to be taken up by tumor cells. Positively charged nanocarriers usually show better permeability than those neutral or negatively charged nanocarriers of the same size (Jiang et al., 2015). The lipid composition of the nanocarrier also largely affects its ability to be internalized. The lipid headgroups, the length of the lipid tail and even the saturability of the lipid carries an impact on the internalization ability of LNPs. Receptor-targeting headgroup shows the best cellular uptake, followed by the cationic amine headgroups. Generally, the longer the lipid tail, the better the displayed uptake of LNPs. Whereas within the same length of the lipid tail, unsaturated lipids demonstrated superior internalization ability (Hare, 1975). Recently, increasing amounts of research reports that the shape of nanocarriers carries some sort of performance effect on its extravasation ability (Jurney et al., 2017; Xie et al., 2017), while the importance of the shape remains unclear.

In our study, we chose phosphatidylcholine (PC) with six different lengths of backbone (C4, C14, C6, C16, C8, C18) to prepare the spherical LNPs (by C14 or C16 or C18 only) or lipid nanostars (LNSs) (by mixing C14:C4 or C16:C6 or C18:C8 at the ratio of 3:1). LNPs synthesized with PC have been reported by numerous articles, whereas the preparation of the LNSs by mixing PC with long and short backbones at certain ratios is first reported in this article. We further investigated the *in vitro* permeability and the *in vivo* extravasation in tumors to reveal the importance of the shape on the uptake of nanocarriers by tumors.

## Materials and Methods

### Materials

1,2-Distearoyl-sn-glycero-3-phosphocholine (18:0 PC; DHPC), 1,2-dipalmitoyl-sn-glycero-3-phosphocholine (16:0 PC; DPPC), 1,2-dimyristoyl-sn-glycero-3-phosphocholine (14:0 PC; DMPC), 1,2-dioctanoyl-sn-glycero-3-phosphocholine (08:0 PC; DOPC), 1,2-dihexanoyl-sn-glycero-3-phosphocholine (06:0 PC; DHPC), 1,2-dibutyryl-sn-glycero-3-phosphocholine (04:0 PC; DBPC), 1,2-dioleoyl-sn-glycero-3-phosphoethanolamine-N-(lissamine rhodamine B sulfonyl) (ammonium salt) (18:1 Liss Rhod PE), 1,2-dipalmitoyl-sn-glycero-3-phosphoethanolamine-N-(lissamine rhodamine B sulfonyl) (ammonium salt) (16:0 Liss Rhod PE), and 1,2-dimyristoyl-sn-glycero-3-phosphoethanolamine-N-(lissamine rhodamine B sulfonyl) (ammonium salt) (DMPE-Rh) were purchased from Avanti Polar Lipids (AL, USA). Phosphate buffer saline (PBS), cell culture media, and fetal bovine serum (FBS) was purchased from Gibco (MA, USA). HepG2 cells were purchased from ATCC (VA, USA). All chemicals and reagents were received and used according to the manufacturer’s protocol.

## Methods

### Synthesis of Various Nanostars

After various combinations of lipid chains at various ratios (data not shown), we successfully synthesized three types of nanostars by using three distinct combinations of lipids (**Table 1**).

**Table 1:** Long chain vs. short chain lipid used in this study and their hydrodynamic sizes and zeta potential.

Long chain lipid	Short chain lipid	Long/short chain lipid ratio	Name	Hydrodynamic size (DLS) (nm)	Zeta Potential (mV)
18 C	8 C	3:1	LNS_18:8_	121.8±13.2	-15.02±3.0
16 C	6 C	3:1	LNS_16:6_	95.0±0.7	-12.4±2.7
14 C	4 C	3:1	LNS_14:4_	81.9±6.0	-12.2±5.3
18 C	–	–	LNP_18_	95.0±0.7	-13.8±1.2
16 C	–	–	LNP_16_	74.4±8.6	-13.8±1.9
14 C	–	–	LNP_14_	72.6±7.4	-12.0±1.5

### Size Distribution and Zeta Potential

The particle size and zeta potential of LNSs and LNPs were determined by dynamic light scattering (DLS) analysis using Malvern Panalytical, MA, USA. The data for each sample were obtained from three replicates.

### Transmission Electron Microscopy Analysis

Five µl aliquots of all LNSs (10 mg/ml) were dropped onto a transmission electron microscopy (TEM) grade carbon-only mesh copper grid. Particles were left on the grid at ambient temperature for 5 min. Each grid was washed five times with distilled water. The specimens were then negatively stained using 2% uranyl acetate and left at ambient temperature for 2 min. Grids were then washed thrice with distilled water and air-dried. The specimens were visualized using a TECNAI F20 electron microscope (Philips Electronic Instruments Corp., Mahwah, NJ).

### Stability of Lipid Nanostars

To determine the stability of LNSs, we synthesized LNSs as mentioned above at 10 mg/ml. The LNSs were then kept in a closed vial at 37°C. At pre-determined time points (1, 2, 4, 8, 12, 24, and 48 h), the size of LNSs were measured by DLS and recorded.

### *In Vitro* Release Profile of Lipid Nanostars

Rhodamine-labeled LNSs (n=3) were dispersed in 1 ml of PBS (pH 7.4) and then transferred to a Float-A-Lyzer G2 dialysis device (MWCO 100 kDa, Spectrum, USA) that was immersed in PBS (pH 7.4) at 37°C. At predetermined intervals (1, 2, 4, 8, 12, 24, 48, 72, 96 h), 5 μl of the NP solution was withdrawn from inside of the dialysis device and mixed with 95 µl of dimethyl sulfoxide (DMSO). After thorough mixing, the fluorescence intensity of rhodamine in each well representing each LNSs was determined by Synergy HT Multi-Mode Microplate Reader (BioTek, USA).

### Cell Culture

The human liver carcinoma cells HepG2 and mice triple negative breast cancer cell line 4T1 were purchased from ATCC and was cultured and used according to the protocols given by the provider. The cells were maintained at 37°C in a humidified cell culture chamber equipped with 5% CO_2_. Cells were maintained in RPMI-1640 medium supplemented with 10% fetal bovine serum (FBS), 100 U/ml penicillin, and 100 µg/ml streptomycin.

### Cellular Toxicity of Lipid Nanostars

To rule out possible cytotoxicity of LNSs toward cells, we performed a cytotoxicity analysis. HepG2 cells were grown at 5,000 cells per well in a 96-well plate. The cells were then treated with LNSs at a concentration range of 0.1–50 mg/ml. LNSs were co-incubated with the cells for 4 h, before washing off with PBS and undergoing further incubation for 48 h. Cell viability was determined *via* alamarBlue assay as previously described (Saw et al., 2017a; Saw et al., 2017b).

### *In Vitro* Cellular Uptake

To visualize the *in vitro* uptake and internalization of nanoparticles, LNSs and LNPs were labeled with fluorescent rhodamine labeled lipid (please see **Table 2**) at 0.5 wt. % of lipid content. HepG2 cells were grown to ~80% confluence on glass coverslips (12 x 12 mm; Fisher Scientific, Texas, USA). Prior to the addition of 200 µg/ml of fluorescently-labeled LNSs or LNPs, the medium was replaced with serum-free medium. After 1 h incubation at 37°C, cells were washed three times with PBS and fixed with 4% (w/v) paraformaldehyde (PFA). Coverslips with fixed cells were mounted onto glass slides with Dako^®^ mounting media and examined using an Olympus Fluoview 1000 confocal microscope (Olympus Imaging Co., Tokyo, Japan). For 3D spheroid culture, HepG2 cells were obtained from monolayer culture using the protocol described previously (Saw et al., 2017a).

**Table 2:** Rhodamine labeled lipid used in LNSs and LNPs for in vitro uptake experiment and in vivo Biodistribution analysis.

Nanoparticles	Rhodamine labeled lipid*
LNS_18:8_	18:1 Liss Rhod PC
LNS_16:6_	16:0 Liss Rhod PC
LNS_14:4_	14:0 Liss Rhod PC
LNP_18_	18:1 Liss Rhod PC
LNP_16_	16:0 Liss Rhod PC
LNP_14_	14:0 Liss Rhod PC

*for in vitro experiments, rhodamine labeled lipid was used at 0.5 wt. %; for in vivo BioD analysis, rhodamine labeled lipid was used at 2 wt. %.

### *In Vivo* Biodistribution Analysis

To visualize the *in vivo* biodistribution of all nanoparticles, LNSs and LNPs were fluorescently labeled with rhodamine labeled lipid (please see Table S1) at 2 wt. % of lipid content. 4T1 cells were subcutaneously implanted into Balb/c mice to generate the xenograft model. Ten milligrams of fluorescently labeled LNSs or LNPs were intravenously *(i.v.)* injected per mouse when the tumor size was around 100 mm^3^. After 24 h, the mice were sacrificed and the major organs, muscle, and tumor were observed by IVIS^®^ Imaging system (PerkinElmer, UK).

### Immunogenicity of Lipid Nanostars After Intravenous Injection

To evaluate the immunogenicity of all nanoparticles, LNSs and LNPs were intravenously injected into Balb/c mice at a dosage of 10 mg per mouse. After 24 h, the mice were anesthetized with isoflurane, and blood was collected and centrifuged at 3,000 rpm for 15 min after standing at room temperature for 15 min. The aspartate transaminase (AST), alanine transaminase (ALT), urea, and creatinine in the serum was analyzed by the Department of Biochemistry, Sun Yat-sen Memorial Hospital.

## Results

### Characterization of the Lipid Nanostars

**Figure 1:**
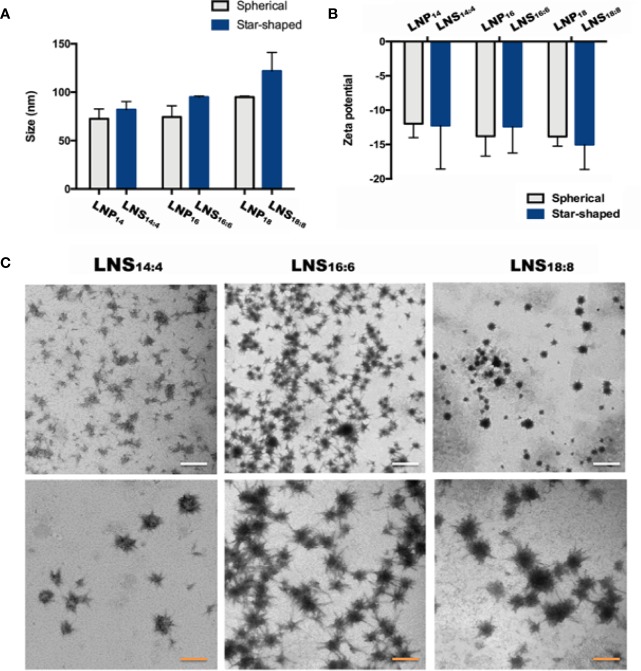
Characterization of the lipid nanostars (LNSs). Size **(A)** and zeta potential **(B)** of the lipid nanoparticles (LNPs) (gray) and LNSs (blue); transmission electron microscopy (TEM) image **(C)** of the LNSs (the write scale bar shows 200 nm, the orange scale bar shows 50 nm).

To investigate the size and surface charge of the LNPs, dynamic light scattering (DLS) was utilized. Despite the shape, the size of six LNPs increased slightly (LNPs: from 72.6 ± 7.4 to 95.0 ± 0.7 nm, LNSs: from 81.9 ± 6.0 to 121.8 ± 13.2 nm) as the length of the lipid backbone increased, however no statistical significance was observed. In addition, the size was similar between LNPs and LNSs when they share the same backbone length (**Figure 1A**). The same trend could be seen in zeta potential results, where the above six LNPs and LNSs were negatively charged (~−12 mV to −15 mV) with no significant difference (**Figure 1B**). The transmission electron microscope (TEM) results showed that the LNS_14:4_, LNS_16:6_, and LNS_18:8_ were star-shaped, and well distributed in water (**Figure 1C**).

### Stability and Release Profile of the Lipid Nanostars

**Figure 2:**
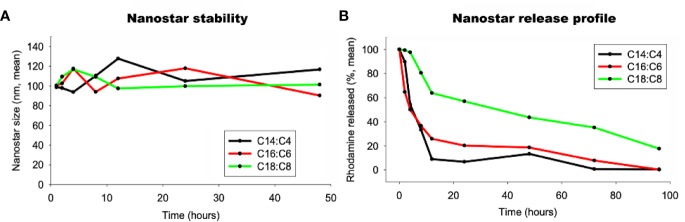
Stability **(A)** and release profile **(B)** of the lipid nanostars (LNSs).

To check if the LNSs were stable, the size of three LNPs were measured by DLS. The diameter of the LNSs was stable at around 100 nm during the first 48 h at 37°C (**Figure 2A**). Then the release profiles of the LNSs were measured at 37°C. As shown in **Figure 2B**, the longer the lipid backbone, the slower the release of the encapsulated rhodamine. LNS_14:4_ rapidly released all the encapsulated rhodamine in 12 h, while ~30% of the encapsulated rhodamine remained in LNS_16:6_ at 12 h and was slowly released in the following 84 h. Moreover, LNS_18:8_ retained ~60% of the encapsulated rhodamine at 12 h, and ~20% was left even at 96 h.

### *In Vitro* and *In Vivo* Uptake of the Lipid Nanoparticles and Lipid Nanostars

**Figure 3:**
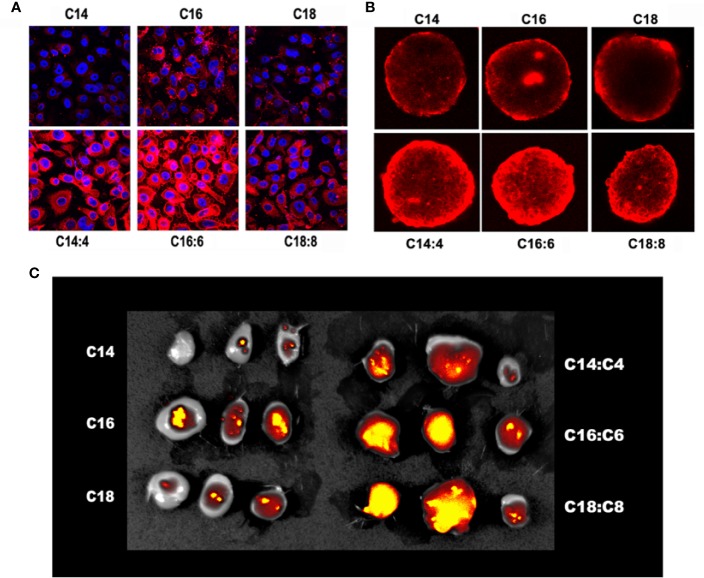
*In vitro* and *in vivo* uptake of the lipid nanoparticles (LNPs) and lipid nanostars (LNSs). The 2D cellular uptake **(A)** of the LNPs (up) and LNSs (down); The 3D cellular uptake **(B)** of the LNPs (up) and LNSs (down); The *in vivo* tumor accumulation **(C)** of the LNPs (left) and LNSs (right); the scale bar shows 50 μm.

To investigate the *in vitro* uptake of the LNPs and LNSs by cancer cells, the human liver cancer HepG2 cells were treated by Rhod-labeled LNPs or LNSs for 4 h, and then observed by confocal laser scanning microscope (CLSM). The results showed that for LNPs, LNP_16_ had the best cellular uptake, while LNP_14_ had the least. However, all the LNSs exhibited significantly enhanced cellular uptake compared with any of the observed LNPs (**Figure 3A**). To further investigate the permeability of the LNPs, HepG2 cells were utilized to measure the uptake in 3D sphere culture. Despite the length of the lipid backbone, the LNPs were mainly accumulated at the outer part of the tumor sphere while LNSs could deeply penetrate into the inner part of the tumor sphere. Still, LNP_16_ showed the best permeability in LNPs, and LNS_16:6_ demonstrated the greatest permeability among the LNSs (**Figure 3B**). To investigate the *in vivo* extravasation in tumors, mice breast cancer 4T1 cells were utilized to build up the xenograft tumor model, and 10 mg of rhodamine-labeled LNPs or LNSs were intravenously injected into each mouse. After 24 h, the tumors were observed by IVIS^®^ Imaging system. Along with the *in vitro* uptake results, LNP_16_ demonstrated the best tumor accumulation among the LNPs, while LNP_14_ performed the least. However, all the LNSs exhibited better tumor extravasation than any of the LNPs, in addition, LNS_16:6_ and LNS_18:8_ showed better extravasation than LNS_14:4_ (**Figure 3C**).

### Biodistribution of the Lipid Nanoparticles and Lipid Nanostars

**Figure 4:**
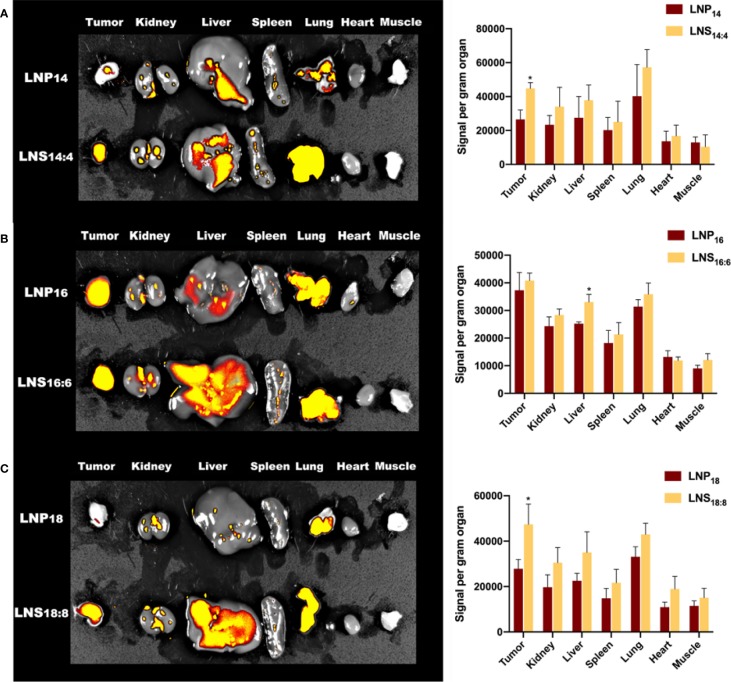
Biodistribution of the lipid nanoparticles (LNPs) and lipid nanostars (LNSs). The image (left) and quantification (right) of *in vivo* biodistribution (BioD) of the LNPs (up) and LNSs (down) prepared by the lipid with backbone of C14/C4 **(A)**, C16/C6 **(B)**, C18/C8 **(C)**. *p < 0.05, compared with relevant spherical control group.

To investigate the biodistribution of the LNPs and LNSs, 10 mg of rhodamine-labeled LNPs or LNSs was intravenously injected into 4T1 tumor bearing mice. After 24 h, the major organs along with the tumor and muscle were observed by IVIS. As **Figure 4** showed, those LNPs mainly accumulated in the liver, kidney, lung, and tumors, while accumulation in other organs was hardly observed. However, LNSs tend to display higher accumulation in the liver and tumors compared with the spherical nanoparticles that share the same lipid backbone length. Especially for LNS_14:4_ and LNS_18:8_, tumor accumulation increased significantly (**Figures 4A, C**). For LNS_16:6_ and LNS_18:8_, liver accumulation increased but does not show statistical significancy (**Figures 4B, C**).

### *In Vitro* and *In Vivo* Toxicity of the Lipid Nanostars

**Figure 5:**
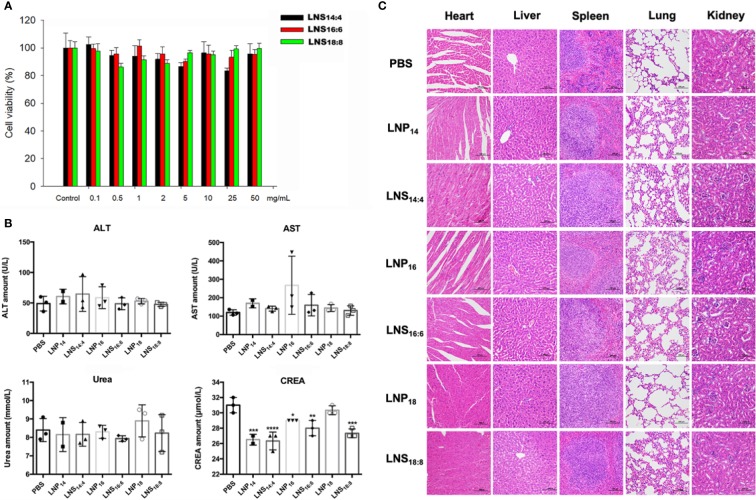
*In vitro* and *in vivo* toxicity of lipid nanostars (LNSs). The cell viability of HepG2 cells treated with different concentrations of the LNSs **(A)**. The serum biochemistry examination of the mice injected with 10 mg of lipid nanoparticles (LNPs) and LNSs **(B)**. The HE staining of the major organs of the mice injected with 10 mg of LNPs and LNSs **(C)**. *p < 0.05; **p < 0.01; ***p < 0.005; ****p < 0.001, compared with PBS group.

To check the *in vitro* safety of the LNSs, HepG2 cells were treated with different concentrations of each LNSs for 4 h followed by 48 h post-incubation before cell viability was measured. As the results showed, although the cell viability values fluctuated slightly, there was no significant difference. Treatment of cells with three types of LNSs revealed that these LNSs were non-toxic over the concentration range of 0.1 mg/ml to 50 mg/ml (**Figure 5A**). To further evaluate the *in vivo* safety, the LNPs or LNSs were injected intravenously at a dose of 10 mg per mouse into Balb/c mice. After 24 h, serum from mice was taken for blood biochemical examination to evaluate their liver and renal function, and the major organs was taken for HE staining to investigate the tissue damage. Compared to the control group, the ALT, AST, and urea values showed no significant changes, whereas the creatinine value of all the groups treated with LNPs decreased significantly, revealing that the liver and renal function of the mice remained normal after injection of LNPs or LNSs (**Figure 5B**). Similarly, the HE staining showed no significant tissue damage of the major organs of LNPs or LNSs treated mice (**Figure 5C**).

## Discussion

PCs is widely used to prepare LNPs (Chakraborty et al., 2015), however not all the PCs are suitable for nanocarrier preparation. In order to prepare qualified nanocarriers, certain lengths of backbone were required (Abumanhal-Masarweh et al., 2019). For example, PC with the backbone length of C14, C16, or C18 were used most frequently (Abumanhal-Masarweh et al., 2019), while those with short backbones (C4, C6, or C8) could hardly form nanoparticles. However, no one has reported whether mixing PCs with different lengths of backbone could be made into nanoparticles. Hence we tried series of fumulation, and finally successfully synthesized three nanoparticles by mixing long backbone PCs (C14, C16, and C18) with short backbone PCs (C4, C6, and C8) at the weight ratio of 3:1. The shape of traditional LNPs are spherical. Surprisingly, when we used PC with different lengths of backbone (C14:C4 or C16:C6 or C18:C8) at the ratio of 3:1 to prepare the nanoparticles, it demonstrated star-like shapes by TEM observation. This might be due to certain amounts of long chain PC being required to stabilize the structure of LNSs, whereas the radial arrangement of short chain PC formed the star-like shape. Due to the slightly increased backbone length of PC, the size increased slightly while no significant change could be seen. As expected, the shape exhibited no significant effect on both the size and zeta potential of the LNSs compared with their spherical controls. Since the samples used for TEM observation were dry whereas the samples were in aqueous solution for DLS detection, the size measured by DLS were bigger than as showed in TEM images due to the layer of hydration around the LNPs (Naha et al., 2015; Kumar et al., 2018).

These LNSs were then studied to investigate their stability and release profiles. As mentioned above, the LNSs prepared by PC with longer backbones exhibited slower release profiles, while the size of all the LNSs remained stable after 48 h at 37°C. This result suggested that the PC with longer chains provided better interweaving with the short chain PC, resulting in stronger inter-molecular interaction. The releasing of the encapsulated rhodamine might be due to the loss of the interwoven short chain PC (C4, C6, or C8) in the LNSs.

In recent years, research regarding the effect of shape on the uptake of nanoparticles has been increasing in popularity. Anisotropic gold nanoparticles with different shapes exhibited significant different cellular uptake (Xie et al., 2017). Similar results were also reported on other inorganic nanoparticles (Karaman et al., 2012; Dai et al., 2015), carbon nanotubes (Smith et al., 2012; Zhang et al., 2017), polymeric nanoparticles (Jurney et al., 2017), and folate nanocarriers (Tahmasbi Rad et al., 2019). Consistent with these findings, our result showed that LNSs obtained significantly enhanced cellular uptake and *in vivo* tumor extravasation ability compared with their spherical controls which shared the same length of lipid backbone. One of the explanations of this phenomenon might be that the radial arrangement of long chain lipids interwoven with short chain lipids constructed arm-like structures around the surface of LNSs. These lipid “arms,” unlike the sphere structures, effectively increased the surface area of the LNSs, thus largely enhancing membrane fusion. Other theories also indicates that the lipid arms of LNSs might increase the odds for it to avoid the steric effect caused by the protein on the cell surface to make direct contact with the cell membrane. Moreover, due to the inherent advantages of lipid nanocarriers, LNSs exhibits lower toxicity than inorganic nanostars meanwhile provide more possibilities to encapsulate therapeutic reagents with different solubility than polymeric nanostars.

Advantages usually come with disadvantages. As illustrated by our biodistribution results, the LNSs experienced increased accumulation in the liver, possibly due to its enhanced surface area. Thus, the question remain whether LNSs were hepatotoxic. However, the results of the serum biochemical examination reassuringly revealed no hepatotoxic effects nor renal toxicity, where the decreased creatinine value suggested enhanced renal clearance. Additionally, increased liver accumulation and renal clearance generally implied worse pharmacokinetic characteristics. However, depending on the pharmacokinetic performance, different formulations could be designed based on its remarkable cellular uptake and *in vivo* tumor extravasation, such as transdermal formulations.

## Conclusion

As a conclusion, we first prepared three LNSs by mixing PC with different backbone lengths (C14:C4 or C16:C6 or C18:C8) at the ratio of 3:1, and then confirmed that they exhibited largely enhanced cellular uptake and *in vivo* tumor extravasation compared with their spherical controls. Among them, LNS_16:6_ showed the best cellular uptake and *in vivo* tumor extravasation, while the characteristics of LNS_14:4_ was increased the most. The enhanced cellular uptake and *in vivo* extravasation might have been induced by the arm-like structures around the surface due to the enlarged surface area of LNSs or the avoidance of the steric effect caused by the protein on the cell surface. Further in-depth investigation is needed to clarify the mechanism, and further research on that topic is being carried out by our team. These LNSs were proven to be safe, similar in size with their spherical controls (~100 nm), and stable at 37°C. The release rate of these LNSs were inversely related to the length of the lipid backbone (LNS_18:8_ showed the slowest release profile, while LNS_14:4_ showed the fastest). Additionally, numerous formulations could be considered depending on the different uptake and pharmacokinetic characteristics displayed by these LNSs, for example injection or transdermal patch. Due to the excellent cellular uptake and *in vivo* tumor accumulation, these LNSs display exciting application potential in the field of tumor therapeutics.

## Data Availability Statement

All datasets generated for this study are included in the article/Supplementary Material.

## Ethics Statement

The animal study was reviewed and approved by Sun Yat-sen Memorial Hospital.

## Author Contributions

PS and XX designed and oversee all experiments. SC and XLiu carried out all experiments. XLi, CL, and WZ helped in *in vivo* experiments, CT and SL analyzed the data, and BL revised and critically analyzed the manuscript.

## Funding

This work was supported by the National Natural Science Foundation of China (81874226 and 81803020), the International Scientific and Technological Cooperation Program from Guangdong Science and Technology Department (2018A050506033), the Thousand Talents Program for Distinguished Young Scholars, the grant from Guangzhou Science and Technology Bureau (201704020131), The Three Million for Three Years Project of SYSMH, Special Funds for the Cultivation of Guangdong College Students’ Scientific and Technological Innovation (pdjh2019a0001) and grant from Guangdong Science and Technology Department (2017B030314026).

## Conflict of Interest

The authors declare that the research was conducted in the absence of any commercial or financial relationships that could be construed as a potential conflict of interest.
